# Genetic Variants in the 3’UTR of *BRCA1* and *BRCA2* Genes and Their Putative Effects on the microRNA Mechanism in Hereditary Breast and Ovarian Cancer

**DOI:** 10.3390/diagnostics10050298

**Published:** 2020-05-13

**Authors:** María Marisela Sánchez-Chaparro, Idalia Garza-Veloz, Omar Alejandro Zayas-Villanueva, Margarita L. Martinez-Fierro, Iván Delgado-Enciso, Mayra Alejandra Gomez-Govea, Laura Elia Martínez-de-Villarreal, Diana Reséndez-Pérez, Iram Pablo Rodríguez-Sánchez

**Affiliations:** 1 Laboratory of Immunology and Virology, Collage of Biological Sciences, Universidad Autónoma de Nuevo Leon, San Nicolas de los Garza, Nuevo Leon 66451, Mexico; marisela.sanchezchp@uanl.edu.mx (M.M.S.-C.); diaresendez@gmail.com (D.R.-P.); 2 Molecular Medicine Laboratory, Human Medicine and HS Academic Unit, Universidad Autonoma de Zacatecas, Zacatecas, Zacatecas 98160, Mexico; idaliagv@uaz.edu.mx; 3 University Center Against Cancer (CUCC), Hospital Universitario “Dr. José E. González”, Collage of Medicine, Universidad Autónoma de Nuevo Leon, Monterrey, Nuevo Leon 64460, Mexico; oa.zayas@gmail.com; 4 Faculty of Medicine, Universidad de Colima, Colima, Colima 28040, Mexico; ivan_delgado_enciso@ucol.mx; 5 Colima State Cancer Institute, Universidad de Colima, Colima, Colima 28040, Mexico; 6 Laboratory of Molecular and Structural Physiology, Collage of Biological Sciences, Universidad Autonoma de Nuevo Leon, San Nicolas de los Garza, Nuevo Leon 66451, Mexico; mayragee@gmail.com; 7 Department of Genetics, Collage of Medicina, Universidad Autonoma de Nuevo Leon, Monterrey, Nuevo Leon 64460, Mexico; laelmar@yahoo.com.mx

**Keywords:** hereditary breast and ovarian cancer, *BRCA1*, *BRCA2*, miRNA, polymorphism, 3’UTR region

## Abstract

Hereditary breast and ovarian cancer (HBOC) syndrome is mainly caused by mutations in the *BRCA1* and *BRCA2* genes. The 3’UTR region allows for the binding of microRNAs, which are involved in genetic tune regulation. We aimed to identify allelic variants on 3’UTR miRNA-binding sites in the *BRCA1* and *BRCA2* genes in HBOC patients. Blood samples were obtained from 50 patients with HBOC and from 50 controls. The 3’UTR regions of *BRCA1* and *BRCA2* were amplified by PCR and sequenced to identify genetic variants using bioinformatics tools. We detected nine polymorphisms in 3’UTR, namely: four in *BRCA1* (rs3092995 (C/G), rs8176318 (C/T), rs111791349 (G/A), and rs12516 (C/T)) and five in *BRCA2* (rs15869 (A/C), rs7334543 (A/G), rs1157836 (A/G), and rs75353978 (TT/del TT)). A new variant in position c.*457 (A/C) on 3’UTR of *BRCA2* was also identified. The following three variants increased the risk of HBOC in the study population: rs111791349-A, rs15869-C, and c.*457-C (odds ratio (OR) range 3.7–15.4; *p* < 0.05). Genetic variants into the 3’UTR of *BRCA1* and *BRCA2* increased the risk of HBOC between 3.7–15.4 times in the study population. The presence/absence of these polymorphisms may influence the loss/creation of miRNA binding sites, such as hsa-miR-1248 in *BRCA1* 3′UTR or the hsa-miR-548 family binding site in *BRCA2*. Our results add new evidence of miRNA participation in the pathogenesis of HBOC.

## 1. Introduction

Breast and ovarian cancer are the most common types of cancer among women, and account for 16% of all cancers in women globally; a total of 1.38 million new cases per year are estimated worldwide [1]. Hereditary cancer syndromes are disorders characterized by an above-average increase in the risk of cancer-specific tissue development [2]. Neoplasms develop because of germ line mutations in specific genes. Among these types of cancer are Li-Fraumeni syndrome, hereditary gastric cancer, von Hippel-Lindau, and hereditary breast and ovary cancer (HBOC) syndromes [2,3,4]. HBOC syndrome is an autosomal dominant disease of incomplete penetrance, with the appearance of neoplasms in the breast and ovary before 40 years of age and is an aggressive form of cancer. Germline mutations in the *BRCA1* and *BRCA2* genes are primarily responsible for HBOC [3,5].

*BRCA1* and *BRCA2* are tumor suppressor genes,and are involved in the mechanisms of double-stranded DNA error repair. In addition to RAD51, p53 is involved in the stability and safety of the genome at cell cycle checkpoints; those that are not repaired send signals of regulated cell death, such as apoptosis and cell arrest. However, errors in the functions of the proteins encoded by these genes provoke cell proliferation [5]. There are regulatory events that reduce the function of *BRCA1* and *BRCA2*, such as promoter hypermethylation and posttranslational regulation by microRNAs (miRNAs) [6,7].

MicroRNAs (miRNAs) are endogenous noncoding RNA molecules of 17–24 nt, capable of fine-tuning transcription of about 50% of genome organisms. They are a type of interfering RNA (iRNA); their target of action is in the 3’UTR regions (≈90%), but they have been found to be able to regulate in sequences located in 5’UTR and in open read frame (ORF) regions (Sun 2013). Sequences of miRNAs, as well as their targets, are highly conserved in species, mainly 7–8 nt in the 5’ sense, called the “seed region”. miRNAs can degrade mRNA completely (perfect complementation in binding site) or repress translation (partial complementation). Because of the high degree of conservation of seed regions and white sites, it has been observed that mutations or single nucleotide polymorphisms (SNPs) at the miRNA binding sites are mechanisms of natural selection of organisms [8]. This mechanism can be observed when SNPs in the 3’UTR regions of various genes are involved in the development of degenerative diseases, such as Tourette’s syndrome, diabetes, cancer in humans, and muscular dystrophies in different vertebrates [9,10].

The northeast region of Mexico is a particular area where the population has a high rate of miscegenation, because of the high migratory flow between mestizo ancestry (characteristics of central and southern Mexico) and a high genetic mixture among the American and European populations (mainly Spanish, Ashkenazi jews, French, and Germans) [11]. SNPs have been identified in 3’UTR *BRCA1* and *BRCA2* gene regions in specific populations. These allelic variants are associated with the increased risk of developing breast or ovary cancer, mainly in those related to HBOC [12,13,14,15]. In addition, SNPs represent a form of trans-regulation of miRNAs that change the mRNA affinity because of the creation or elimination of binding sites, which could result in highly inheritable mutations that produce the phenotypes associated with the development of HBOC. We performed an exploratory study in a Mexican population in order to evaluate allelic variations in the 3’UTR regions of the *BRCA1* and *BRCA2* genes, and to determine if their presence allows for the onset and/or elimination of miRNA binding sites, and whether the mutations are associated with HBOC syndrome.

## 2. Materials and Methods

### 2.1. Study Population

A case-control study was performed. Electronic records of patients previously treated at the University Cancer Center of the University of Nuevo Leon were selected based on specific inclusion criteria (women with clinical data of HBOC: diagnosis of primary breast cancer, ≤45 years of age at the time of diagnosis, and familiar history of breast or ovarian cancer in ≥3 relatives). After selection, the patients were contacted. Clinically healthy women with a risk estimation <1% using the Gail calculation tool, ≤45 years of age, were included as controls. The women who agreed to participate in the study were interviewed for updating the clinical record and signed consent. Individuals belonged to the Northeastern region of Mexico, including ancestry in the first and second degree. The protocol was submitted and approved by the Ethics Committee of the College of Medicine of the Universidad Autonoma de Nuevo Leon (ID: G15-003).

### 2.2. Amplification of 3′UTRs BRCA1 and BRCA2 Regions

DNA was isolated and purified from peripheral blood using the commercial kit DNA Isolation, Serum and Plasma (Qiagen, Frederick, MD, USA), following the manufacturer’s specifications. The quality of the samples was verified using a spectrophotometer (Nanodrop, Thermo Scientific™, Whaltam, MA, USA). The genomic material was concentrated to 50 ng/µL, eluted to a final volume of 100 µL, and stored at −20 °C until PCR amplification. The primers were designed for the specific 3′UTR of each gene of interest, using the sequences provided by GenBank for *BRCA1* (NM_007294.3) and *BRCA2* (NM_000059.3), and Primer Blast software was used to obtain an amplified product (AP) of 1420 bp for *BRCA1* (forward: ACCTGATACCCCAGATCCCC; reverse TTTGGAAGTGTTTGCTACCAGG) and 902 bp BRCA2 (forward: GAACAGGAGAGTTCCCAGGC; reverse: AATCAGTGCCAATTTGAAAGCA). A PCR reaction was performed using JumpStart Taq ReadyMix ™ (Sigma-Aldrich™, St. Louis, MO, USA). The reactions had a final volume of 30 µL, 10 µM of which was used for each primer, and 1 µL was for the DNA template (50 ng/µL). The final volume was completed with nuclease-free water. The amplification program consisted of one cycle of 94 °C × 4 min, followed by 35 amplification cycles (denaturation at 94 °C × 1 min; alignment at 60 °C × 30 s; and extension at 72 °C × 1 min), and a final extension cycle of 72 °C × 6 min. To clean the AP, we added 2 mL of ExoSAP-IT. ^®^ Product Cleanup (Affymetrix, Santa Clara, CA, USA) to 5 µL of AP, and incubated at 37 °C × 15 min and then 80 °C × 15 min to inactivate the reagent. Because of the lack of DNA samples from individuals, these procedures could not be analyzed in duplicate in all cases.

### 2.3. Sequencing and Analysis of 3′UTR Regions

The 3′UTRs region labelling was performed using the BigDye Terminator v3.1 Cycle Sequencing commercial kit (Applied Biosystem, Foster, CA, USA). Because the resolution of the capillary is approximately 500 bp, we designed internal primers to amplify short fragments (350–550 bp) for a better reading accuracy (Table 1).

The sequencing reaction, with 1 µL of BigDye, 2 µL of 10× buffer, 1 µL of oligonucleotide (30 ng/µL), 3.5 µL of AP, and milliQ water, was prepared to a final volume of 10 µL. The labelling program consisted of one cycle of 96 °C × 1 min, and 25 cycles of 96 °C × 10 s, 50 °C × 5 s, and 60 °C × 4 min. The resulting products were purified, stored, and protected from light at −20 °C. We used the ABI-3130xl sequencer Avant Genetic Analyzer (Applied Biosystems), a 36-cm capillary (Applied Biosystem) and the POP-4 polymer, following the manufacturer’s instructions. After sequencing, the files that contained electropherograms were analyzed using the GeneStudio™ Professional software v20.

The sequences obtained after testing were stored in FASTA format for further analysis. The sequencing results were compared with the wild type sequences of the *BRCA1* and *BRCA2* genes. The relationship between the known variants and the risk of HBOC were verified using the online bioinformatic and supports programs Ensemble Genome Browser (ENSEMBL; http://www.ensembl.org/), the Human Gene Mutation Database (HGMD; http://www.hgmd.cf.ac.uk/ac/all.php), and Human Genome Variations Society (HGVS; http://www.hgvs.org/dblist/dblist.html). We also used the NCBI database of SNPs (dbSNP) (http://www.ncbi.nlm.nih/gov/SNP) to obtain information about genetic variations.

### 2.4. Clinical and Statistical Analysis

Simple frequency calculations of the HBOC risk variables and the clinical characteristics of the study participants were determined by direct counts, and it were expressed as percentages and means ± standard deviation. An evaluation of the possible relationship between genotypes/alleles and HBOC was done using the SNPstat program (https://www.snpstats.net/start.htm) from the Institute of Oncology of Catalonia (Catalonia, Spain) [15]. Odds ratios (ORs) were estimated for each genotype variant. The Hardy–Weinberg equilibrium (HWE) was analyzed using online software. In the case control studies, tests for deviation from the Hardy–Weinberg equilibrium, and tests for association, (https://ihg.gsf.de/cgi-bin/hw/hwa1.pl; Neuherberg, Germany) *p*-values < 0.05 were considered significant.

## 3. Results

### 3.1. HBOC Population

A total of 50 patients were clinically characterized with HBOC and, therefore, they were included in the study; the mean age at the first diagnosis of breast cancer was 37.28 years (range 25–45 years). Of these patients, 34% had a status at least double or triple negative (TNC) at estrogen (ER), progesterone (PR), and human epidermal growth factor receptor 2 (HER2/neu) receptors ((ER–PR–HER2+ *n* = 3; ER+ PR–HER2– *n* = 8; TNC *n* = 17). The clinical relevance of the carriers of mutations in 3′UTR *BRCA1* and *BRCA2* are shown in Table 2.

**Figure 1:**
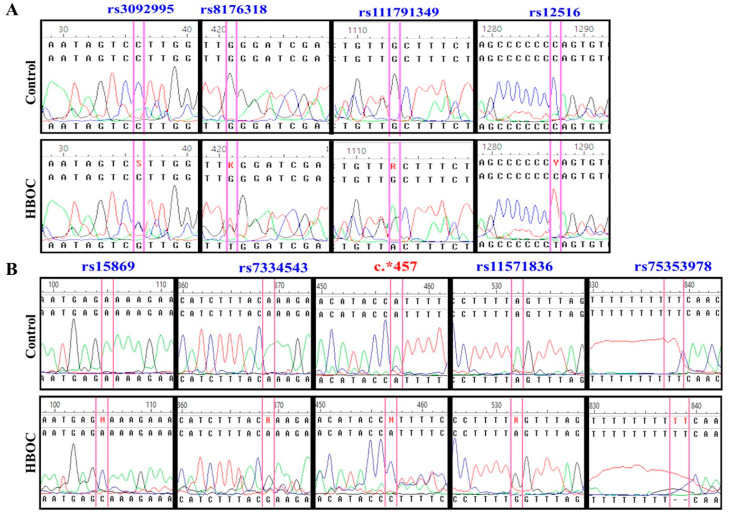
Identification of variants’ single nucleotide polymorphisms (SNPs). Allelic variants of each 3’UTR region were identified by comparing the alleles carrying the HBOC and healthy patients; blue legends indicate SNP previously reported; red legend indicate a new genetic variant. (**A**) Comparison of 3’UTR of *BRCA1*; (**B**) Comparison of 3’UTR of *BRCA2.*

The purpose of our study similar to that proposed by Erturk et al. (2014) [12], who carried out an exploratory study on the genetic situation of the population,and will require future case-control trials with a larger number of samples. We identified nine SNPs in the 3′UTR region of *BRCA1* and *BRCA2* genes. These variants were rs3092995 (C/G), rs8176318 (C/T), rs111791349 (G/A), and rs12516 (C/T) for 3′UTR *BRCA1*. For *BRCA2*, we identified the polymorphisms rs15869 (A/C); rs7334543 (A/G); rs1157836 (A/G); rs75353978 (TT/del TT); and a new variant, not identified yet, in position c.*457 (A/C) (Figure 1). The genotypic and allelic frequency percentages are shown in Table 3.

### 3.2. Hardy-Weinberg Equilibrium (HWE) and Allelic Risk

Based on the information obtained, we performed a test for the balance of the Hardy-Weinberg equilibrium, with the purpose of determining if there was independence between the observed alleles and what was expected. The SNPs that were considered for the following studies were rs8176318 and rs12516 for *BRCA1*, and rs15869 and variant c.*457 for *BRCA2*. In the remaining SNPs, HWE was unbalanced for the control group. Although the most common cause is the genotyping method, we consider that the causes were due to the high migratory flow of the area and its genetic consequences. A significant difference between the groups was found with homozygous minor frequent alleles (MAF) of the SNPs rs111791349 (allele A, *p* = 0.047), rs15869 (allele C, *p* = 0.032), and c.*457 (allele C, *p* = 0.007), which were considered as a risk for HBOC patients (Table 4 and Table S1). 

### 3.3. Presence of Genetic Changes Could Modify miRNA Binding Mechanisms

**Figure 2:**
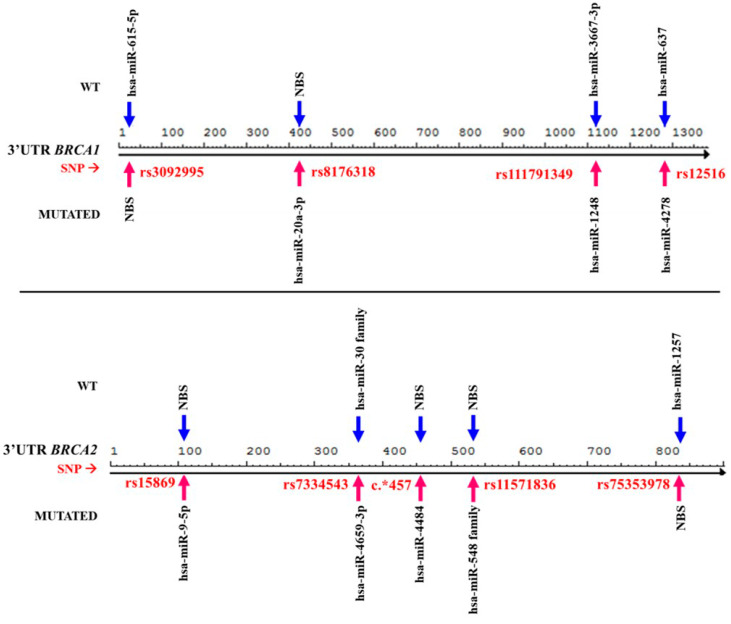
Comparative map of miRNAs bound between normal (WT) and mutated sequences. Trans modifications can be observed in the miRNA mechanism because of the presence of allelic variants. Arrow blue indicate miRNAs binding site in WT; Arrow red indicate miRNAs bind site into mutations. NBS—site with no miRNA binding site.

We determined the effect of the allelic changes in the 3′UTR regions of the *BRCA1* and *BRCA2* genes on the miRNA binding mechanism. Our aim was to establish if the miRNA seed region was maintained and if it was not modifying binding with its usual mRNA, or conversely, if the allelic changes provoked the elimination of recognition sites or created new binding loci. In silico analysis showed that there are trans modifications in miRNA regulatory mechanisms (Figure 2). The changes allow different for miRNAs, considered as oncomiRs, i.e., hsa-miR-1248 and -4278 for *BRCA1* 3′UTR, or the creation of members of the miRNA family binding site, such as hsa-miR-548 in *BRCA2* (Table 5).

### 3.4. Trans-Mechanisms miRNAs Affect Molecular and Cellular Regulation

A comparative molecular ontology and cellular pathway analysis between the miRNAs that lost their binding sites and the new ones that did bind because SNPs were performed. Previous experimental evidence indicates that there are changes in the metabolic and gene signaling pathways of differentiated controls for each group of miRNAs. The pathways involved in the generation of molecules were characteristic of tumor microenvironments, such as adherents binding, hormonal signaling, hypoxic environments, etc. This was also the case for molecular pathways of genome stabilization and regulation at the cell cycle checkpoints and cell arrest. This proves that the presence of allelic variations in patients modifies the molecular and cellular pathways. For example, miRNAs that bind to normal genotypes regulate the mechanisms of lysine degradation (*p* = 0.0006), HIF-1 signaling (*p* = 0.0118), and the central carbon of cancer metabolism (*p* = 0.0119), simultaneously. However, this regulation ceases to be performed in the presence of other miRNAs. These apparent regulatory control differences suggest that both early tumor development and cancer progression could be indicative of aggressive cancer phenotypes in carrier allelic variants patients.

## 4. Discussion

We observed that more than 50% of patients with HBOC were diagnosed at the disease stage (IIB and IIIA). The Northeastern region of Mexico is one of the areas with the highest morbidity and mortality rates due to breast cancer over 20 years (28.58:100,000), compared with the national average statistics (18.97:100,000) [16,17]. A fundamental analysis in the confirmatory diagnosis of breast cancer is the determination of the expression status of membrane receptors; because of the degree of cancer heterogeneity, these analyses help to determine the prognosis and specific treatment. In addition, oncological treatments should consider specific molecular therapeutic targets. For example, ER^+^ and PR^+^ patients are eligible for tamoxifen treatment. With the introduction and approval of trastuzumab (Herceptin), over-expressing HER2/neu patients are candidates for its use.

In previous reports, it was observed in central Mexico that 23–25% of HBOC patients were double or triple negative for these receptors. However, in our study, it was observed that about 53% had at least double negatives (36% TNC and 16% double negative), surpassing almost double what was expected [17]. These characteristics are associated with aggressive neoplasia and poor prognosis. This work can be used as an exploratory study of the genetic characteristics of HBOC patients in the region, based on the specific characteristics, allelic and genotypic frequencies, and the new allelic variant identified (c.*457) with a strong statistical relationship.

Molecularly, miRNAs related to the regulatory changes by 3’UTR mutations of both *BRCA1* and *BRCA2* are related in the mechanisms of neoplastic development. These changes presume a change in metabolic and cellular function, which must be validated by means of luciferase assays in cell cultures [12,18,19,20]. In the larger study of 3’UTR *BRCA1* in five sample collections, the variants c.* 528 (G/C), c.*718 (A/G), c.*1271 (T/C), c.*309 (T/C), c.*379 (G/A), c.*823 (C/T), and c.*264 (C/T) decreased the 3’UTR activity in luciferase assays, while variants c.* 291C>T and c.* 1139G>T increased the activity. However, none of these were considered pathological [21,22]. Rs3092995 SNP (c*36 C/G) was reported in 1998, and the CG and GG alleles were observed in breast cancer cases of African American patients (28%). OR adjusted by age was 3.5 (95% CI, 1.2–10.0); the authors determined that this variant is in a partial linkage imbalance. We found rs3092995 SNP in both the HBOC cases and the controls (18% and 16%, respectively); the like study above explained, rs3092995 was not balanced in the mestizo population of Northeastern Mexico [11].

Our modified results were like those from the analyzes performed by García et al. (2016) in the subgroup of patients of the French national study GENESIS (GENE SYSters), in which patients with family breast cancer were evaluated, with at least one sister diagnosed. They detected in their population the variants c.*421 (G/T) (MAF = 0.324) and c.* }1287 (C/T) (MAF = 0.342) for the 3’UTR *BRCA1*, which were also present in our study. In 3’UTR-*BRCA2* variants c.* 105 (A/C)(MAF = 0.161), c.*369 (A/G) (MAF = 0.222), and c.*532 (A/G) (MAF = 0.197) were screened. All the variants were found in patients of Caucasian ethnicity. Historically, the Northeastern region of Mexico has been characterized by a population dynamic in which many people have ancestors in the third or fourth generation from Europe or the Middle East; this could explain the presence of this genetic vestige like a founder mutation [23]. We found a new genetic variant in 3′UTR *BRCA2*, c.*457 (A/C), which has not been described so far in any other similar studies. This is relevant, because the *C* allele presents a risk in HBOC patients (*p* = 0.007). This finding is important, given the few mutations described in the 3’UTR *BRCA2.*

Rs-8176318, -12516, and -15869 SNPs were previously found and analyzed in a Chinese population, and they evaluated SNPs’ role in the miRNA mechanism; rs15869 demonstrated a suppression of the *BRCA2* expression in MCF7 and MDA-MA-231 cell lines through luciferase assays. The authors suggest that this interaction increases the risk of breast cancer. This can support part of our results, but we should perform similar assays [23,24].

Control of bias in case-control studies is essential. We performed each process in this study with strict quality control and bias in case-control selection, data collection, experiment design and operation, data entry, and the results of statistical analysis. However, a source of possible bias may be because most cases come from the hospital, and this can lead to Berkson’s bias. We reduced this type of bias by evaluating only pathologically confirmed cases of newly diagnosed breast cancer patients, and data were collected through the questionnaire through personal interviews. Confusion bias was effectively controlled by strict inclusion criteria and matched controls. However, some limitations in the present study cannot be ignored. The relatively small sample size reduces statistical power, especially in some subgroups.

On the other hand, a factor that may be affecting the HWE of both populations (and the main reason for carrying out this genetic study) is that the Northeastern region of Mexico has characterized from the beginning of the 20th century to date by a high settlement of European populations (Spanish, Ashkenazi Jews, French, and Germans), Middle and Far East (Saudi Arabia, China, Japan, and Korea), and American (due to the border with the United States). Likewise, because of the economic wealth of the region (Monterrey is the city with the highest quality of life index in Latin America), there is a continuous flow of mestizo population from central and southern Mexico, which is genetically different [11]. These reasons make the gene flow dynamics of this region high, and therefore these alleles are on their way to achieve genetic equilibrium. We proposed that some allelic variants may be due to founder genes from all the communities mentioned.

Future findings could be complemented by determining the expression levels of the set of miRNAs presumably being deregulated by mutations in the 3’UTR regions, as well as validating deleted/created miRNA binding sites using assays of the luciferase activity and precipitation of Dicer, among others, in various types of cancer cell lines. It would also be of great importance to establish whether the mutations are germinal and whether they can be traced in groups of families with histories of breast and ovarian cancer, as well as prostate cancer.

With respect to our study, because of the number of sequenced individuals, the statistical findings cannot be extrapolated to the population of Northeastern Mexico (approximately 11.51 million people). However, this work provides preliminary data for the study of specific genetic variants for the 3’UTR regions of *BRCA1* and *BRCA2* genes in this population, the linked miRNAs, and their implication in the fine tuning of the genome.

## 5. Conclusions

Genetic variants in 3’UTR of *BRCA1* and *BRCA2* increased the risk of HBOC by 3.7–15.4 times in the study population. The presence/absence of these polymorphisms may influence the lost/creation of miRNA binding sites, such as hsa-miR-1248 in *BRCA1* 3′UTR or the hsa-miR-548 family binding site in *BRCA2*. Our results provide new evidence of the miRNAs’ participation in the pathogenesis of HBOC. However, molecular predictions should be evaluated by a luciferase activity assay or similar assays to demonstrate the deep impact of the allelic variations found in fine molecular regulation. The genetic alterations found and analyzed can support further research that may clarify its effect on *BRCA1* and *BRCA2* genes molecularly and clinically. Future studies should be defined to evaluate miRNAs binding mechanism in order to develop diagnostic, prognostic, and treatment tools based on miRNAs technology. Thus, we consider this exploratory study as a pioneer in the understanding of HBOC in a Mexican mestizo population.

## Figures and Tables

**Table 1:** Internal primers used for labelling sequences. Primer used in to perform 3′UTR (*BRCA1* and *BRCA2*) sequencing.

Gene	Primer Sequences 5′→3′	Tm (°C)
*BRCA1*	ACCTGATACCCCAGATCCCC	60.0
	ATCCAAGCACTCTCCTTCC	57.3
	CCTGTGTTCACAAAGGCAGA	62.0
	TGATCTTGGCTCACTGCAAC	57.3
	GGCAGGAGAATCACTTCAGC	57.9
	GCAACAGCTTCCTTCCTGGTGGG	56.1
	GGAAAATGAAACTAGAAGAGATTT	58.0
	AGGCTCTGAGAAAGTCGGCT	60.0
*BRCA2*	GAACAGGAGAGTTCCCAGGC	56.0
	CCCACCTCAGCTTCTCAAAG	57.3
	GGTGGCTCATGCCTGTAATC	57.9
	TTGCTCAAAAGGAAACACCA	56.1
	CAGTTATTTGATGCAGATTCC	58.0
	AATCAGTGCCAATTTGAAAGCA	60.0

**Table 2:** Clinical description of the study population. Clinical aspects of hereditary breast and ovarian cancer (HBOC) participants.

Clinical Data	>%	Clinical Data	%
**Diagnostic age (years)**	Mean 37.28 ± 3.30	**Receptor status (ER, PR, HER2/neu)**	
**BMC (kg/m^2^)**	Mean 27.76 ± 5.59	ER^+^ PR^+^ HER2^+^	10%
**Disease free-survival**		ER^+^ PR^+^	40%
>36 months	29%	ER^+^ HER2^+^	2%
<36 months No data	21% 50%	HER2^+^	6%
**Menarche**	11.77 ± 2.48	ER^+^	4%
**Menopause**		Triple negative	34%
Yes	38%	**Therapy**	
No	62%	Chemotherapy	
**Oral-contraceptive use**		AC(4)-Taxol(12)	28%
Yes (>5 years)	74%	Anthracyclines	18%
No	20%	Taxanes	10%
**Inclusion criteria**		Capecitabine	2%
Family history	41%	Not especified	10%
Diagnostic >40 years	59%	No chemotherapy	32%
**Births**		Radiotherapy	
0	20%	Adjuvant	76%
1,2	54%	Palliative	2%
3–5	20%	Adjuvant/Palliative	2%
>5	3%	No radiotherapy	16%
**Screening method**		Hormone therapy	
Autoexploration	88%	Yes (Tamoxifen)	54%
Clinical finding	2%	No hormone therapy	42%
Mastography	4%	**TNM Score**	
**Smoke**		I A	4%
Yes	78%	I B	10%
No	22%	II A	16%
**Histology**		II B	20%
Infiltrating ductal	74%	III A	34%
Medullar carcinoma	4%	III B	4%
Lobullar	2%	III C	4%
Ductal/Lobullar	2%	IV	6%
**Grade of cell differentiation**		**Metastasis**	
G1	6%	Bone	10%
G2	28%	Visceral	2%
G3	36%	Bone, visceral	2%
Gx	28%	Bone, visceral, lymph node	2%

**Table 3:** Comparison of the genotypic and allelic frequencies of variants between the HBOC and control groups by gene.

Gene	Variant	HBOC Patients					Control			
		Genotype Frequency			MAF		Genotype Frequency			MAF
*BCRA 1*	rs3092995	C/C	C/G	G/G	G		C/C	C/G	G/G	G
		82%	0%	18%	18%		76%	8%	16%	20%
	rs8176318	C/C	C/T	T/T	T		C/C	C/T	T/T	T
		84%	10%	6%	11%		90%	10%	0%	5%
	rs111791349	G/G	G/A	A/A	A		G/G	G/A	A/A	A
		68%	12%	20%	26%		76%	18%	6%	15%
	rs12516	C/C	C/T	T/T	T		C/C	C/T	T/T	T
		82%	4%	14%	16%		84%	16%	0%	8%
*BCRA 2*	rs15869	A/A	A/C	C/C	C		A/A	A/C	C/C	C
		84%	6%	10%	13%		82%	18%	0%	9%
	rs7334543	A/A	A/G	G/G	G		A/A	A/G	G/G	G
		94%	0%	6%	6%		94%	0%	6%	6%
	c.*457	A/A	A/C	C/C	C		A/A	A/C	C/C	C
		66%	12%	22%	28%		68%	30%	2%	17%
	rs11571836	A/A	A/G	G/G	G		A/A	A/G	G/G	G
		82%	4%	14%	16%		82%	14%	40%	11%
	rs75353978 **	TT/TT		delTT			TT/TT		delTT	
		32%		68%	32%		92%		8%	8%

* MAF = Minor Allele Frequency; ** rs75353978 SNP is a deletion of TT bases, it is indicated like delTT.

**Table 4:** Tests for association between genetic variants and hereditary breast and ovarian cancer. All tests were carried out with a 95% confidence interval (CI).

Gene	Variant/Alleles	Risk Allele 1					Risk Allele 2			
		Allele Freq. Difference	Heterozygous	Homozygous	Allele Positivity		Allele Freq. Difference	Heterozygous	Homozygous	Allele Positivity
		[1]↔[2]	[11]↔[12]	[11+]↔[22]	[11]↔[12+22]		[2]↔[1]	[22]↔[12]	[22]↔[11]	[11+12]↔[22]
*BRCA1*	rs8176318 (C/T)	2.4 (0.8–7.0)	1.1 (0.3–4.0)	7.5 (0.4–149.4)	1.7(0.5–5.7)		0.4 (0.1–1.3)	0.1 (0.01–3.5)	0.1 (0.01–2.7)	0.1 (0.01–2.7)
		0.118 (P)	0.918	0.078	0.372		0.118 (P)	0.1185	0.078	0.079
	rs12516 (C/T)	2.2 (0.9–5.4)	0.3 (0.05–1.3)	15.4 (0.9–277.7)	1.2 (0.4–3.3)		0.5 (0.2–1.1)	0.02 (0.001–0.5)	0.1 (0.004–1.2)	0.1 (0.003–1.0)
		0.082 (P)	0.078	**0.001**	0.79		0.082 (P)	**0.001**	**0.0099**	**0.006**
*BRCA2*	rs15869 (A/C)	1.5 (0.6–3.7)	0.3 (0.08–1.3)	10.7 (0.6–200.5)	0.9 (0.3–2.5)		0.7 (0.3–1.6)	0.03 (0.001–0.8)	0.1 (0.005–1.7)	0.1 (0.004–1.51)
		0.366 (P)	0.097	**0.032**	0.79		0.366 (P)	**0.005**	**0.032**	**0.0218**
	c.*457 (A/C)	1.9 (0.96–3.8)	0.4 (0.1–1.2)	11.3 (1.4–92.8)	1.1 (0.5–2.5)		0.5 (0.3–1.0)	0.04 (0.004–0.4)	0.1 (0.01–0.7)	0.1 (0.01–0.6)
		0.063 (P)	0.096	**0.007**	0.832		0.063 (P)	**0.0005**	**0.007**	**0.0021**

Cell contents: odds ratio (95% confidence interval) and p-value. rs8176318: Allele [1] = T; allele [2] = C, rs12516: allele [1] = T; allele [2] =C, rs15869: allele [1] = C; allele [2] = C; c.*457: allele [1] = C; allele [2] = A. Significative values are indicate in bold.

**Table 5:** In silico effect of allelic variants on the miRNAs-binding seed regions. Some SNPs modified the sequence regions of the mRNA causing the deletion or creation of miRNA binding sites. Red line indicates a new miRNA binding site created; red cross indicates that a miRNA binding site were deleted.

Gene	Variant	microRNA	ΔΔG (Kcal/mol)	Duplex SNP-miRNA		Effect
*BRCA1*	rs8176318 (G/T)	hsa-miR-20a-3p	Wt: 0.00	miRNA:	3′-gaaauuCACGAGU-AUUACGUCa-5′	**Created**
					|||:||| ||||||	
			SNP: −22.70	UTR:	5′-aaccctGTGTTCACAAATGCAGa-3′	
	rs12516 (C/T)	hsa-miR-4278	Wt: −18.30	miRNA:	3′-guucccGUUUGGGGGAUc-5′	**Created**
					:|: |||||||	
			SNP: −21.60	UTR:	5′-cttcccTAGCCCCCCTAg-3′	
		hsa-miR-637	Wt: −28.30	miRNA:	3′-ugcgucucGGGCUUUCGGGGGUCa-5′	**Eliminated**
					||| |: ||||X||	
			SNP: −16.00	UTR:	5′-taggtcttCCCTAGCCCCCCTAGt-3′	
*BRCA2*	rs15869 (A/C)	hsa-miR-9-5p	Wt: −14.00	miRNA:	3′-ucuuugguuaucUAGCUGUAUga-5′	**Created**
					|| :|| |	
			SNP: −23.20	UTR:	5′-ttatgttgcacaATGAGCAAAga-3′	
	c.* 457 (A/C)	hsa-miR-4484	Wt: −11.10	miRNA:	3′-ACCCCGAAGAGGGCGGAAAA-5′	**Created**
					:| ||||||	
			SNP: −23.70	UTR:	5′-TGAAATAAACATACCCTTTT-3′	

## Supplementary Materials

The following are available online at https://www.mdpi.com/2075-4418/10/5/298/s1, **Table S1:** Tests for deviation from Hardy-Weinberg equilibrium and tests for association.
