# Supplementary material for: Genetic Variants in the 3’UTR of BRCA1 and BRCA2 Genes and Their Putative Effects on the microRNA Mechanism in Hereditary Breast and Ovarian Cancer
Source: Diagnostics (Basel). 2020 May 13;10(5):298. doi: 10.3390/diagnostics10050298 (PMC7277914; doi:10.3390/diagnostics10050298)
Supplement: Supplementary file 1 [file diagnostics-10-00298-s001.pdf]

Supplementary Table 1. Tests for deviation from Hardy-Weinberg equilibrium and tests for association

Gene	Allele	Frequencies	Cases %	Controls %	P-value (cases vs control)	OR	95% I. C.
3'UTR *BRCA1*	rs3092995 (c.*36 C > G)	**Genotype**	n = 50	n = 50			
		CC	82	76	0.7184	Reference	
		CG	0	8	0.04282	0.103	0.005-1.978
		GG	18	16	0.93779	1.043	0.365-2.979
		**P-value HWE**	1.537E-12	1.137E-07			
		**Allele**					
		C	0.825	0.805			
		G	0.175	0.195			
	rs8176318 (c*421 G > T)	**Genotype**	n = 50	n = 50			
		GG	84	90	0.1178	Reference	
		GT	10	10	0.91771	1.071	0.289-3.967
		TT	6	0	0.07813	7.494	0.376-14.9405
		**P-value HWE**	0.0005	0.7097			
		**Allele**					
		G	0.893	0.952			
		T	0.107	0.048			
	rs111791349 (c*1113 G > A)	**Genotype**	n = 50	n = 50			
		GG	68	76	0.05402	Reference	
		GA	12	18	0.60965	0.745	0.240-2.311
		AA	20	6	0.04856	3.725	0.946-14.671
		**P-value HWE**	1.14E-06	0.0375			
		**Allele**					
		G	0.854	0.745			
		A	0.146	0.255			
	rs12516 (c*1287 C > T)	**Genotype**	n = 50	n = 50			
		CC	88	88	0.08172	Reference	
		CT	4	16	0.07816	0.07816	0.051-1.279
		TT	14	0	0.00996	1.5361	0.850-27.764
		**P-value HWE**	1.76E-09	0.5386			
		**Allele**					
		C	0.845	0.922			
		T	0.155	0.078			

Gene	Allele	Frequencies	Cases %	Controls %	P-value (cases vs control)	OR	95% I. C.
3'UTR *BRCA2*	rs15869 (c*105 A > C)	**Genotype**	n = 50	n = 50			
		AA	84	82	0.36601	Reference	
		AC	6	18	0.09686	0.325	0.082-1.288
		CC	10	0	0.03152	10.741	0.576-20.045
		**P-value HWE**	2.04E-07	0.4843			
		**Allele**					
		A	0.874	0.912			
		C	0.126	0.088			
	rs733453 (c*369 A > G)	**Genotype**	n = 50	n = 50			
		AA	94	94	1.0000	Reference	
		AG	0	0	1.0000	1	0.019-5.144
		GG	6	6	1.0000	1	0.192-5.210
		**P-value HWE**	1.54E-12	1.54E-12			
		**Allele**					
		A	0.943	0.943			
		G	0.057	0.057			
	c*457 (A > C)	**Genotype**	n = 50	n = 50			
		AA	66	68	0.06251	Reference	
		AC	12	30	0.09596	0.412	0.143-1.191
		CC	22	2	0.00645	11.333	1.385-92.772
		**P-value HWE**	6.81E-07	0.6555			
		**Allele**					
		A	0.725	0.833			
		C	0.275	0.167			
	rs11571836 (c*532 (A > G)	**Genotype**	n = 50	n = 50			
		AA	82	82	0.30085	Reference	
		AG	4	14	0.11308	0.286	0.056-1.458
		GG	14	4	0.11308	3.5	0.686-17.864
		**P-value HWE**	1.76E-09	0.0439			
		**Allele**					
		A	0.845	0.893			
		G	0.155	0.107			
	rs75353978 c*838-839 (TT > - -)	**Genotype**	n = 50	n = 50			
		TT	92	32	2.32E-18	Reference	
		- -	8	68	6.38E-10	2.4438	7.495-7.968
		**P-value HWE**	1.54E-12	1.54E-12			
		**Allele**					
		TT	0.326	0.923			
		- -	0.674	0.077			
